# Considering microRNAs as measures of extrinsic domains of risk in the context of underlying genetic characteristics: type 2 diabetes as an exemplar

**DOI:** 10.3389/fgene.2026.1934355

**Published:** 2026-09-18

**Authors:** Benjamin M. Stroebel, Alexis Jimenez, Karla J. Lindquist, Dara Torgerson, Kayla D. Longoria, J. Cesar Ignacio-Espinoza, Elena Flowers

**Affiliations:** 1 Department of Physiological Nursing, School of Nursing, University of California, San Francisco, CA, United States; 2 Keck Graduate Institute, Claremont, CA, United States; 3 Department of Obstetrics, Gynecology, and Reproductive Sciences, University of California, San Francisco, CA, United States; 4 Institute for Human Genetics, University of California, San Francisco, CA, United States; 5 Department of Epidemiology and Biostatistics, University of California, San Francisco, CA, United States; 6 Cizik School of Nursing, University of Texas, Houston, TX, United States

**Keywords:** disease etiology, genetic admixture, microRNAs, risk prediction, social determinants of health

## Abstract

The etiology of most health conditions is complex, arising from a combination of risk factors that include both intrinsic (i.e., genetic characteristics) and extrinsic (e.g., behaviors, the environment, social factors) domains. Until relatively recently, health science research typically conflated the biological construct of genetic ancestry with the social constructs of race and ethnicity. The advent of ancestry informative markers (AIMs) and related statistical methods marked an important advance, enabling the quantification of genetic ancestry independent of the constructs of race and ethnicity. Another type of molecular marker (microRNAs) has shown potential utility for quantifying extrinsic domains of risk, including behavioral, environmental, and social factors, within the context of underlying genetic characteristics. Given these markers offer distinct yet complementary information, combining them may provide a more rigorous and comprehensive approach to evaluating the combined domains of intrinsic genetic risk with extrinsic risk factors to improve accuracy in prediction of risk for complex diseases like type 2 diabetes (T2D).

## Introduction

The etiology of most health conditions is complex, arising from both intrinsic (i.e., genetic characteristics) and extrinsic (e.g., behaviors, environment) risk factor domains. As opposed to single gene testing, whole-genome approaches have made it feasible to describe a wider spectrum of inter-individual variability in intrinsic genetic characteristics. These more comprehensive approaches are essential for understanding multifactorial genetic risk for complex conditions (e.g., type 2 diabetes) and related complications. Polygenic risk scores (PRS) that can be derived from genome-wide association studies (GWAS) describe the combined risk from numerous genetic loci, often with small individual effect sizes, and provide an approach to describing overall intrinsic risk for health outcomes for an individual. However, when applied to risk prediction models, PRS provide limited improvement in accuracy compared to models that include only demographic, clinical, and family history characteristics (Flannick and Florez, 2016; Padilla-Martínez et al., 2020).

A well-documented limitation of many early GWAS is the lack of study samples that are globally representative (Fatumo et al., 2022). This limitation has important implications for genetic association studies. Genetic characteristics vary based on global continental ancestry with some genetic variants being highly present in some groups compared to others. The consequence of the lack of global ancestral representation in study samples is that genetic risk for a given health outcome is only captured for the ancestral haplotypes that are included in a study sample. This creates a “we do not know what we do not know” scenario in which latent confounding results in some individuals being erroneously categorized as low risk only because genetic risk in their ancestral background is unknown. An approach to address the problem of latent confounding by ancestry is through the evaluation of ancestral markers, which measure geographic ancestral origin based on the concept of linkage disequilibrium (Yang et al., 2005). First through the HapMap project (International HapMap Consortium, 2003) and now more commonly using the 1,000 genomes project (1 KG) (Genomes Project Consortium et al., 2015) and other developing reference databases, individual ancestry can be measured and operationalized as a quantitative variable for statistical modelling. Applied to a group, shared ancestral origins of a cohort of individuals can be defined as population substructure.

While ancestral markers are useful for leveraging intrinsic genomic data to estimate genetic ancestry and associated disease risk, there are extrinsic domains of risk that are also significant contributors. Until relatively recently, health science research typically conflated the biological construct of ancestry with the social constructs of race and ethnicity. The advent of ancestral markers and statistical methods to leverage these characteristics improved on this shortcoming by providing a way to quantify biological ancestry, discrete from the social constructs of race and ethnic group categorization.

Building on whole genome technologies, there are now methods for measuring the degree to which immutable genetic characteristics are manifested in a given individual and how these processes relate to risk for health outcomes. This includes measurement of the transcriptome, proteome, metabolome, and epigenome, among others. MicroRNAs (miRs) are small (18–24 nucleotide) molecules that regulate the process of translation of messenger RNAs (mRNAs) to amino acids (Flowers et al., 2013). Regulation by miRs is one process by which an organism can rapidly modify its physiological function to survive its environment. Changes in expression levels of miRs in response to behaviors and exposures have been observed over relatively short time periods. For example, changes in expression of skeletal muscle miRs (e.g., miR-1, miR-133, miR-206) before and after a 30-min treadmill stress test have been used to evaluate cardiac function (Flowers et al., 2015). In addition to these dynamic properties, circulating miRs found in blood are relatively less susceptible to degradation compared to other RNA molecules and can be obtained through standard minimally invasive venipuncture that does not require specialized and costly collection and storage procedures, making them promising candidates for future translation as biomarkers of disease risk. Numerous prior studies across many disease conditions have provided evidence to suggest that miRs may provide both predictive and mechanistic insights about risk for health outcomes, including type 2 diabetes, which is one of the leading causes of mortality and morbidity in the US and of the highest public health priority (Flowers et al., 2021; Flowers et al., 2022a; Asam et al., 2023; Flowers et al., 2023; Kariuki et al., 2023; Flowers et al., 2024; Stroebel et al., 2025; Lewis et al., 2024).

Given their dynamic properties and functional role in the manifestation of interactions between intrinsic genetic characteristics and extrinsic risk factors, miRs may provide an opportunity to quantify the impact of these interactions. For example, miRs may provide information about the impact of exposure to racism in the context of underlying genetic ancestry, further differentiating these two constructs as discrete domains of risk for health outcomes (Flowers et al., 2022b). We found only one prior study that evaluated for differences in miR expression between groups defined by distinct genetic ancestry (ancestral groups), which reported that 107 individual miRs were differentially expressed between Utah residents with Northern and Western European ancestry (CEU) and Yoruban Nigerian (YRI), suggesting the possibility for differences in miR expression that are derived from the underlying genome (Huang et al., 2011).

The purpose of this paper is to provide a perspective on the application of miRs as biomarkers for the cumulative impact of risk factors for complex diseases, summarize potential methods for evaluating miR expression patterns in the context of ancestry, and to demonstrate how this information might be used for describing risk, using type 2 diabetes as an exemplar. Future studies might leverage ancestry-associated miR signatures to differentiate this domain of intrinsic risk from potentially modifiable extrinsic exposures in a cumulative model of risk for complex diseases.

### Methods to integrate MiR profiles with genetic ancestry

Given the paucity of studies that have evaluated associations between miR expression and genetic ancestry, consensus about the best approaches for characterizing miR expression profiles in the context of genetic ancestry has yet to be established. Broadly, supervised methods can be employed to describe sets of miRs that are associated with defined ancestral representation, while unsupervised methods can be used to first define observed patterns of miR expression and then determine if they are related to ancestral characteristics. With either approach, pre-processing of miR data based on the assay used for detection will be required (e.g., filtering, normalization, control for batch effects) as well as adjustment for multiple comparisons and internal validation. Methods that might be considered for identifying these miR signatures are briefly described below.

Penalized regression methods, including ridge regression, lasso regression, and elastic net regression, provide a supervised approach for evaluating associations between multiple correlated miRs and measures of genetic ancestry. These methods account for multicollinearity through coefficient penalization, reducing overfitting and improving model stability. Ridge regression shrinks coefficients while retaining all predictors, lasso performs variable selection by shrinking some coefficients to zero, and elastic net combines both approaches for correlated predictors. These methods can be applied using either continuous or categorical measures of genetic ancestry to identify ancestry-associated miR signatures.

Canonical linear discriminant analysis (CLDA) identifies linear combinations of miRs, known as linear discriminants, that provide the best separation between predefined ancestral groups. By reducing multiple correlated miRs into a smaller number of discriminant functions, CLDA can improve classification efficiency while facilitating interpretation of miR patterns associated with discrete ancestry categories.

Hierarchical clustering is an unsupervised method that groups participants based on similarities in miR expression patterns without requiring predefined ancestry categories. The resulting clusters can subsequently be evaluated for associations with continuous measures of genetic ancestry or categorical ancestry groups to determine whether distinct miR expression patterns correspond to underlying genomic characteristics.

Consensus clustering is an unsupervised approach that evaluates the stability of participant clusters across repeated resampling procedures. Following feature reduction to remove low variance miRs, consensus clustering identifies stable patterns of miR expression and provides metrics to guide selection of the optimal number of clusters. Participant cluster membership can then be tested for associations with categorical ancestry groups or continuous measures of genetic ancestry.

Principal component analysis (PCA) reduces the dimensionality of correlated miR predictors by combining them into a smaller number of principal components. In supervised PCA, outcome information (e.g., population substructure) is incorporated during component construction to improve prediction, whereas unsupervised PCA derives components solely from variation in miR expression. Component loadings can subsequently be examined to determine the relative contribution of individual miRs, and the resulting components can be tested for associations with genetic ancestry and other extrinsic factors.

## Exemplar study

### Design and methods

We leveraged data from our prior study that evaluated miR expression in a subset (n = 854) of participants from the Diabetes Prevention Program (DPP) trial (Flowers et al., 2023; Flowers et al., 2024) to assess relationships between miRs and genetic ancestry in people at risk for type 2 diabetes. The DPP trial primary endpoint was incident type 2 diabetes at 2 years; complete sample characteristics, trial design, and methods have been described in detail in previous publications (The Diabetes Prevention Program Research Group, 2000; Diabetes Prevention Program (DPP) Research Group, 2002). All data reported here were collected from the baseline enrollment visit for the DPP trial. MiR data were collected from plasma following the manufacturer’s protocol using the Firefly Bioworks Multiplex Circulating MicroRNA Assay as previously described (Flowers et al., 2023) and are available through a data repository (Flowers, 2024). Briefly, 58 miRs were measured from banked plasma based on preliminary data, and following the manufacturer’s guidelines, quality control filtering measures included expression levels > 2x the lower limit of detection (58 miRs retained); miR expression >0 in >90% of samples (35 miRs retained); and miRs with >80% median absolute deviation (28 miRs retained). Genetic data for the sample were obtained through controlled access to dbGaP (accession number phs000681.v2.p1).

ADMIXTURE (Version 1.3.0) was used to derive individual-level proportions of ancestry, leveraging genotype data aligned with 1 KG reference populations (International HapMap Consortium, 2003). The ggplot2 package (R, 2022) was used to visualize ancestry proportions as stacked bar blots for assessment of ancestry at both the individual and population level (Flannick and Florez, 2016; Padilla-Martínez et al., 2020; Fatumo et al., 2022; Yang et al., 2005).

To illustrate one potential analytical workflow, we applied unsupervised k-means consensus clustering to identify miR expression patterns before analyzing their association with measures of genetic ancestry. Prior to analysis, all miR values were standardized using z-scores. Optimal cluster count across k = 1 through k = 5 was evaluated by consensus matrices, cumulative distribution functions, delta area plots, and item tracking plots, and optimal number of clusters was determined based on the most favorable number across these metrics. Random forest models were then used to determine the relative importance of individual miR expression in determining assignment to each cluster. All cluster analyses were performed using R (2023).

Histograms of proportion ancestry were visually assessed to determine normality of distribution. Differences in ancestry proportions between miR clusters were assessed with Kruskal–Wallis test with adjustment for multiple comparisons by the Bonferroni method. Cox proportional hazards models adjusted for trial arm were used to test for associations between ancestry and incident type 2 diabetes over 2-years.

## Results

Based on similar cross-validation (cv) error values for k = 5 (cv = 0.56) and k = 8 (cv = 0.56) and to facilitate mapping to the 1 KG super-populations in this exploratory analysis, we retained five ancestral groups that characterize this sample. These mapped to European (k = 4), African (k = 2), Amerindigenous (k = 1), East Asian (k = 3) and South Asian (k = 5).

A total of 28 miRs were included in consensus clustering. Based on evaluation of cluster counts across all metrics, we selected k = 4 clusters (average consensus cluster score 0.89). Expression of miRs 146a, 24, 221, 23a, 342, 27a, 92a, 486, 20b, 21, 17, 126,7c, 29b, and 192 were most important in determining cluster assignment (Figure 1). European (p < 0.0001) and African (p < 0.0002) ancestry were significantly different across the four miR consensus clusters after correction for multiple comparisons (Figure 2).

**FIGURE 1:**
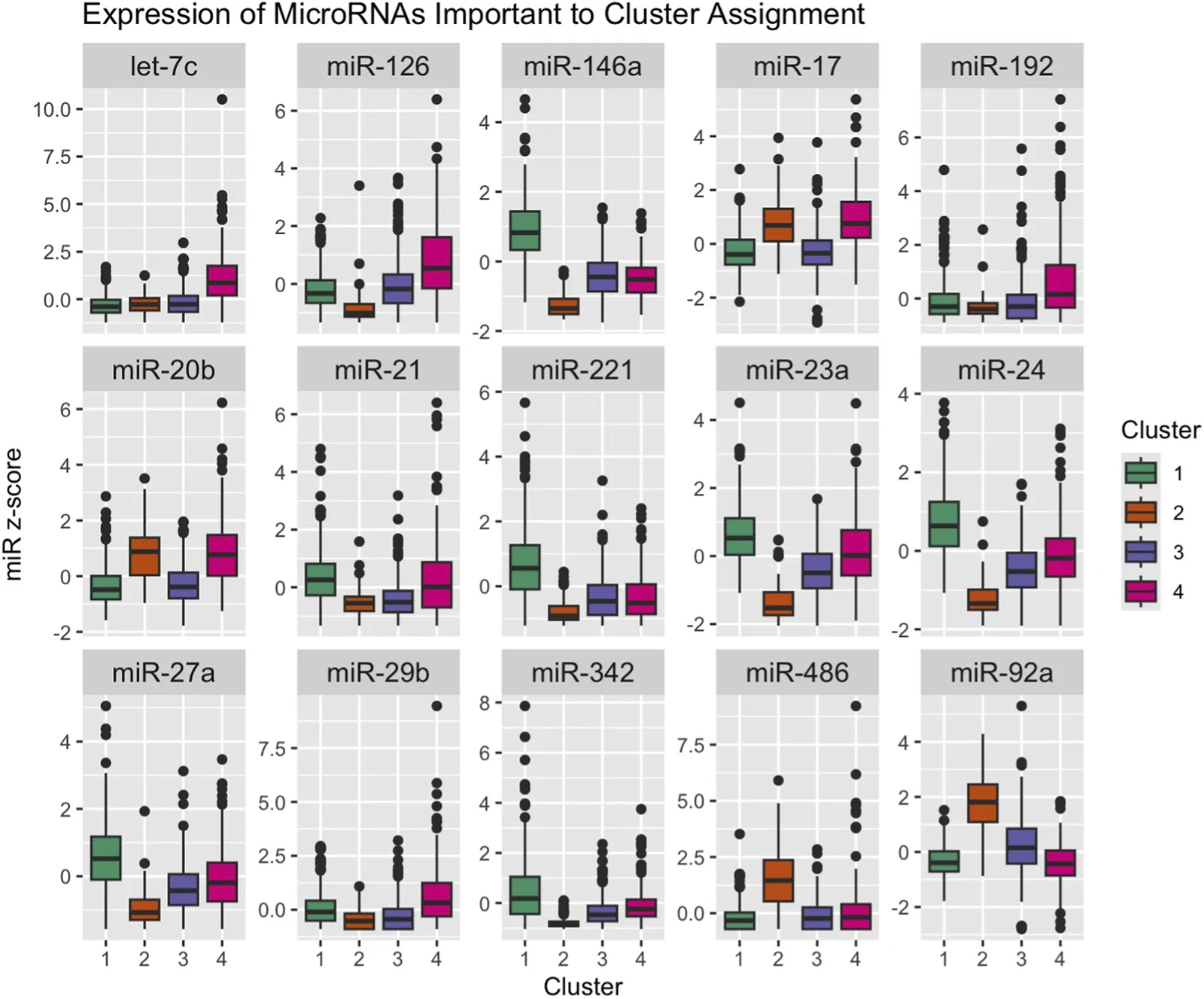
Distribution of MicroRNAs that Determined Consensus Cluster Assignment. Box whisker plots show distributions of microRNAs that determined cluster assignment. The central horizontal line indicates the median; the lower and upper bounds of the box represent the 25th and 75th percentiles, respectively. Whiskers extend to the minimum and maximum data points within 1.5 times the interquartile range. Individual points plotting beyond the whiskers indicate outliers.

**FIGURE 2:**
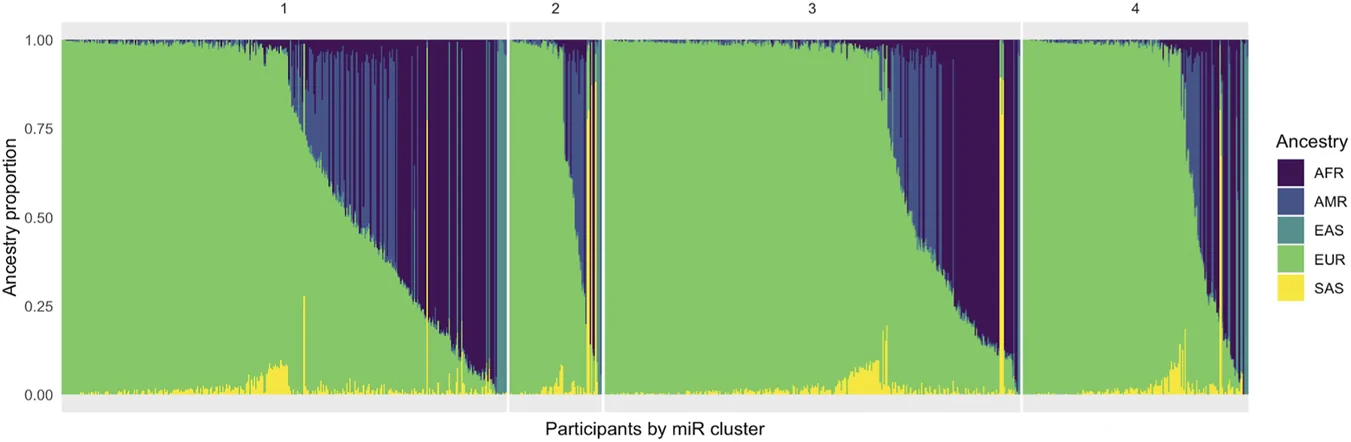
Ancestry proportions for participants from the Diabetes Prevention Program trial stratified by microRNA consensus cluster. Vertical bars represent an individual’s proportion of ancestry inferred from the 1 KG reference database. AFR–African; AMR - Amerindigenous; EAS–Eastern European; EUR–European; SAS–South Asian.

For miR consensus clusters, there were no significant differences in incident type 2 diabetes at 2-years, but among those treated with risk-reduction interventions who showed significant differences in type 2 diabetes incidence at 2-years compared to placebo, cluster one was associated with increased odds (OR 3.06 (1.10, 9.88)) in a behavioral intervention group and decreased odds (OR 0.43 (0.18, 0.97)) in a medication group. No ancestries were significantly associated with incident type 2 diabetes over 2-years.

## Discussion

Health science research has historically conflated race and ethnic categories with continental genetic ancestry. While these two constructs are related and sometimes correlated, they are distinct. This is evidenced by the more complex and continuous biological genetic structure as compared to discrete socially defined categories of race and ethnicity, the latter of which is how most study samples have previously been characterized. Among many major contributions, the Human Genome Project facilitated measurement of genetic ancestry using AIMs based on the principle of linkage disequilibrium, and newer technologies now capture an even broader spectrum of ancestry-linked genetic variation. This presents the opportunity to control for continuous genetic ancestry, which is representative of individual differences in background genetic variation, including loci that may be associated with disease risk. This is distinct from socially assigned race and ethnic groups that may function as a proxy for social experiences that affect disease risk; however, within these groups, there is still significant heterogeneity in extrinsic exposures and risk for type 2 diabetes. There remains the major challenge of measuring the biological impact of heterogeneous social experiences. MiRs exhibit dynamic expression and have the potential to overcome existing challenges by providing a method to quantify the biological consequences of extrinsic factors that may affect disease risk. However, miRs are encoded by the underlying genome, and are likely to show differences in presence and function based on individual genetic characteristics, including ancestry. Our prior study in this same sample showed that miRs are significant predictors of incident type 2 diabetes (Flowers et al., 2023), supporting the idea that they characterize risk resulting from both intrinsic genetic and extrinsic factors. We suggest that miRs may be a way to disentangle intrinsic genetic risk for disease that is linked to genetic ancestry from extrinsic domains of risk that are linked to race and ethnicity.

We identified only two prior studies (Flowers et al., 2022b; Alimena et al., 2024), including our own from the same dataset of participants from the DPP trial that considered miR expression differences between race and ethnic groups to determine if the construct of race and related social experiences might impact miR expression patterns. However, neither study controlled for genetic ancestry to distinguish the distinct impact of social experiences from ancestry-informed prevalence of genetic loci associated with disease risk. As highlighted in our previous paper (Flowers et al., 2022b), failing to control for these underlying ancestral characteristics may be one reason for lack of validation and inadequate generalizability of research findings across studies.

This exemplar study showed admixture within the study sample, which was recruited from across the US, with relatively smaller representation of ancestry groups that map to Amerindigenous, East Asian, and South Asian regions. There were differences in the proportion of European and African ancestries across miR clusters (Figure 2). This supports the idea that ancestry is associated with miR expression patterns, which are in part influenced by underlying genetic loci and polymorphisms. Each individual will have certain miR characteristics that are predefined by their underlying genome, and these are partially represented by the miR consensus clusters. However, the variability in representation of ancestry proportion across miR clusters also supports the hypothesis that miRs have the potential to capture factors from extrinsic domains of risk, including socially defined race and ethnic groupings, that are superimposed upon underlying genetic characteristics. Differential associations between miR clusters and prevalent type 2 diabetes among participants treated with behavioral and pharmaceutical risk-reduction interventions further supports the potential that miRs are candidate biomarkers that can capture individual-level heterogeneity in risk in response to extrinsic factors.

The data presented in this paper are intended to show proof of concept and have limitations. The 1 KG ancestry reference database is not representative of all ancestral groups, and our sample also had limited representation of some ancestries (i.e., Amerindigenous, East Asian, South Asian), which may have limited power to test for differences in proportions. The sample is limited to people with prediabetes versus the full spectrum of diabetes risk, which decreased power to test associations with a complex disease outcome. Optimal approaches for miR clustering have not been established, and future studies are needed to compare performance of the methods presented above and in Supplementary Table S1. We propose that the combination of ancestral markers to estimate genetic ancestry in combination with miRs, which are readily detectable in peripheral circulation and have previously been shown to have dynamic changes in response to extrinsic risk factors, may support the design of studies that can more precisely assess causal relationships between underlying intrinsic and relatively immutable genetic characteristics compared to physiological responses to extrinsic factors that are dynamic over time. Future studies can incorporate measurements of ancestry with polygenic and miR risk scores to determine if this improves accuracy in risk prediction, and, within these models, determine if miRs capture risk from extrinsic factors. With larger sample sizes, ancestry-specific polygenic and miR risk scores may also be tested. The ultimate application of these molecular and statistical methods will be to improve accuracy of risk prediction for complex diseases like type 2 diabetes and to characterize combinations of intrinsic and extrinsic exposures that may be mechanistic targets for risk reduction.

## Data Availability

Publicly available datasets were analyzed in this study. This data can be found here: https://doi.org/10.7910/DVN/MJDBJE https://dbgap.ncbi.nlm.nih.gov/beta/study/phs000681.v2.p1/#study.
