# Supplementary material for: Considering microRNAs as measures of extrinsic domains of risk in the context of underlying genetic characteristics: type 2 diabetes as an exemplar
Source: Front Genet. 2026 Sep 18;17:1934355. doi: 10.3389/fgene.2026.1934355 (PMC13631256; doi:10.3389/fgene.2026.1934355)
Supplement: Supplementary file 1 [file DataSheet1.docx]

| **Cluster 1** | **Cluster 2** | **Cluster 3** | **Cluster 4** |
| --- | --- | --- | --- |
| **miR-146a^2,3^** | **miR-92a** | **miR-146a^1,2^** | **let-7c** |
| **miR-24^3^** | **miR-486** | **miR-24^1^** | **miR-17** |
| **miR-221** | **miR-146a^1,3^** | **miR-23a^1,2^** | **miR-20b^3^** |
| **miR-23a^2,3^** | **miR-23a^1,3^** | **miR-20b^4^** | **miR-126** |
| **miR-342** | **miR-126** | **miR-21** | **miR-29b** |

The 5 miRs most important to assignment in each cluster, listed in order of relative importance. Superscript numerals denote other clusters to which a miR is important for assignment
